# Early experience with uniportal robotic thoracic surgery lobectomy

**DOI:** 10.3389/fsurg.2022.1005860

**Published:** 2022-09-23

**Authors:** Edoardo Mercadante, Nicola Martucci, Giuseppe De Luca, Antonello La Rocca, Carmine La Manna

**Affiliations:** Thoracic Surgery Unit - Istituto Nazionale Tumori â€“ IRCCS â€“ Fondazione G. Pascale, Naples, Italy

**Keywords:** robotic thoracic surgery, rats, uniportal RATS, thoracoscopic surgery, VATS, uniportal VATS, robotic thoracic surgery (RATS)

## Abstract

**Background:**

Invasiveness is considered one of the cornerstones of every field of surgery, and video-assisted thoracoscopic (VATS) approaches are now routinely used worldwide to perform pulmonary resections. Recently, robotic-assisted thoracic surgery (RATS) has become the preferred technique in many centers; it is routinely performed using three or four ports with at least one service incision, contrasting with the real concept of invasiveness, especially when compared to uniportal VATS (U-VATS). Hereby, we present our early experience with uniportal RATS (U-RATS) pulmonary resections for early-stage lung cancer. Technical details of surgical steps are accurately described and commented on.

**Results:**

Twenty-four consecutive patients with lung cancer underwent U-RATS anatomical pulmonary resections at our institute. All procedures were completed with the uniportal approach. The mean operative time was 210 min (range 120–350); in the last 10 cases, the operative time was significantly reduced (180 min) compared to the first 10 cases (232 min) (*p* < 0.02), showing a very fast learning curve. The postoperative pain score was comparable to that for U-VATS and was constantly low.

**Conclusions:**

U-RATS is a safe and feasible technique, combining the advantages of U-VATS with the well-known advantages of robotic surgery.

## Introduction

Invasiveness is considered one of the cornerstones of every field of surgery due to less morbidity and faster postoperative recovery compare to open surgery. Video-assisted thoracoscopic (VATS) approaches are now routinely used worldwide to perform pulmonary resections and are not only limited to standard procedures or early-stage lung cancer but also in the case of advanced stages requiring complex reconstructions (1–4). In particular, since 2004, uniportal VATS (U-VATS) has progressively gained relevance in the thoracic surgery units, including our center, due to its invasiveness compared to multiportal approaches, without differences in feasibility and oncological outcomes (5, 6). Recently, robotic-assisted thoracic surgery (RATS) is increasingly becoming the preferred technique in many centers worldwide. The main advantages are the 3D vision in the operative field, the intuitive management, and the easy maneuverability, allowing safer and more accurate surgical acts due to the wristed arms and the use of bipolar energy and grasping in both hands (7, 8). However, RATS is routinely performed using three or four ports with at least one service incision (9) in contrast to the real concept of less invasiveness. The possibility of blending the uniportal approach with robotic technology would be an enormous improvement in terms of feasibility, safety, oncological outcomes, and enhanced postoperative recovery. An update of the literature during the revision process of our paper showed a very recent description of the technique (10, 11) and a previous case report (12). Thus, considering our personal experience with U-VATS and standard robotic techniques, we recently started our U-RATS program. Herein, we present our early series of U-RATS pulmonary resections for early-stage lung cancer, focusing on feasibility, safety, surgical technique, and early postoperative outcomes.

## Patients and methods

Based on our experience with U-VATS and four-port robotic surgery, in January 2022 at the IRCCS “G. Pascale Foundation” National Cancer Institute of Naples, we started the U-RATS program. Twenty-four consecutive patients (9 males and 15 females, mean age 64 ± 11 years) with lung cancer underwent anatomical pulmonary resections. All patients signed a standard informed consent form as this approach does not have an experimental purpose. Patient characteristics are reported in Table 1. Standard preoperative workup was performed including routine blood examinations, pulmonary function tests, arterial blood gas analysis, cardiological assessment, total body computed tomography (CT), and total body positron emission tomography (PET). In most patients, whenever possible, a preoperative diagnosis of lung cancer was achieved by CT fine needle biopsy or fiberoptic bronchoscopy; in other cases, the diagnosis was intraoperatively confirmed after wedge resection. Our standard pain control for minimally invasive surgery includes intraoperative nerve blocking of 3–4 intercostal spaces with 100 mg of local anesthesia (Ropivacain) performed at the beginning of surgery, followed by intravenous postoperative Ketorolac 90 mg/24 h for 2 days, plus 1 g of paracetamol if needed in selected cases. No opioids are routinely used. All surgical procedures have been performed at the console by the same surgeon. In this report, we focus on surgical technical steps, feasibility, and early postoperative outcomes, including pain evaluation using the Numeric Pain Rating Scale (NRS), complications, and functional recovery, evaluated during the outpatient visit through specific questions about life activities.

**Table 1:** Patients’ characteristics.

Pts	Sex	Age	Tumor size (cm)	Lesion location	Smoking	Comorbidities
1	M	78	3.2	Right lower lobe	Ex	Ischemic heart disease, hypertension
2	F	68	0.9	Left lower lobe	No	Hypertension
3	M	67	2.3	Left lower lobe	Yes	Hypertension, COPD
4	F	57	1.2	Left upper lobe	Yes	Hypertension
5	F	66	2.0	Right upper lobe	Yes	Hypertension, COPD,
6	M	68	2.0	Left upper lobe	Yes	COPD
7	F	47	1.6	Right upper lobe	Yes	Hyperthyrodism
8	M	77	1.3	Left upper lobe	Yes	Hypertension
9	F	78	2.5	Right upper lobe	No	Vasculopathy
10	F	58	2.0	Right middle lobe	Ex	Hypertension
11	F	60	1.2	Left lower lobe	Yes	
12	M	79	1.7	Right lower lobe	Yes	Ischemic heart disease, hypertension, COPD
13	F	43	0.8	Right lower lobe	Ex	
14	M	66	2.2	Right upper lobe	Yes	Hypertension, COPD
15	F	62	1.8	Left lower lobe	Yes	COPD
16	F	56	1.6	Left upper lobe	No	
17	M	60	2.8	Right upper lobe	Ex	Diabetes
18	M	79	2.4	Left lower lobe	Ex	Hypertension
19	F	47	1.5	Right middle lobe	Ex	
20	F	80	3.0	Left upper lobe	Yes	Hypertension, COPD
21	F	70	1.3	Right upper lobe	Ex	Hypertension, diabetes
22	M	53	1.8	Left lower lobe	Yes	Hypertension
23	F	62	2.4	Right lower lobe	Ex	Hypertension
24	F	53	1.5	Left lower lobe	Ex	

## Surgical technique

**Figure 1:**
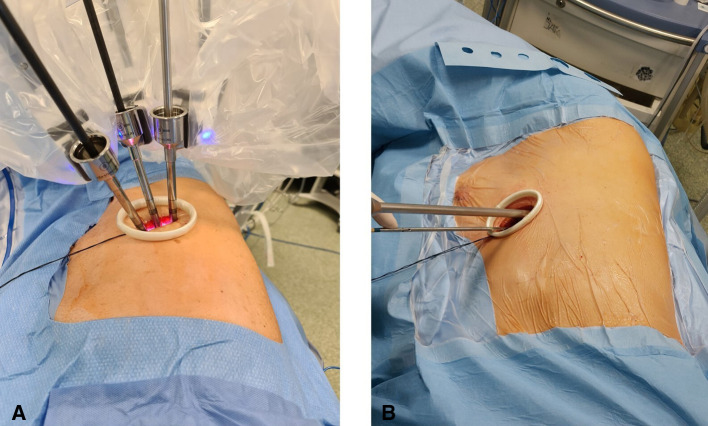
(**A**) U-RATS: incision in the middle axillary line with trocars perpendicular to the target. (**B**) U-VATS: incision by the anterior approach with trocars tangential to the target.

All procedures were performed under general anesthesia with single-lung ventilation using the *da Vinci Xi* robotic surgical system. The patient is placed in lateral decubitus like a posterolateral incision and flexed to expose the intercostal space better. A 4-cm skin incision is made at the V or VI intercostal space in the middle axillary line. The correct location of the incision is of paramount importance, and it can vary based on the target of surgery and the chest shape. The incision must be as close as possible to the vascular structures that must be resected. This allows the robotic arms to be perpendicular to the target, limiting their conflict and optimizing the available space (Figure 1A). A soft wall protector is used to avoid excessive trauma to the chest wall.

**Figure 2:**
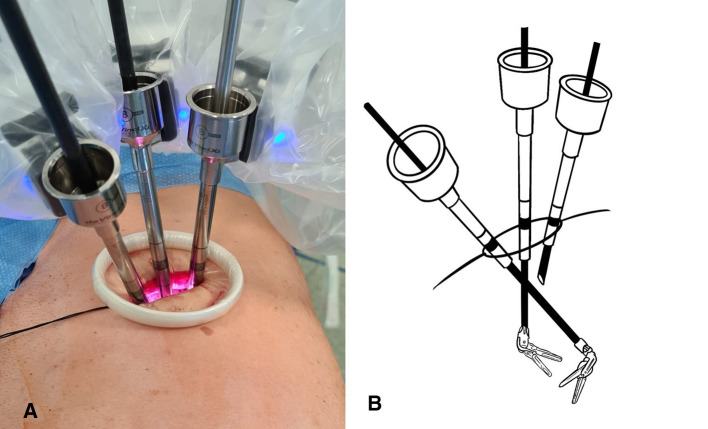
(**A**) External trocars vision. (**B**) Vision of instruments crossing inside the chest.

Three robotic arms are always used, and the trocars are directly anchored to the arms without any pressure valve. A 30-degree 10-mm camera is placed on the posterior edge of the incision as in U-VATS surgery, and the other two arms are placed in the remaining space anteriorly. The operative robotic arms work by crossing each other inside the chest; thus, the right robotic arm will be the left surgeon's hand and the left robotic arm will be the right surgeon's hand, as shown in Figure 2. With this setting, to avoid the mirroring effect, it is necessary to apply a reverse mode to the console touchpad, allowing the right hand to control the left robotic arm and vice versa. Gauze peanuts are freely inserted in the chest to be used to mobilize the lung, reducing parenchymal trauma and optimizing movements. A robotic Maryland bipolar forceps dissector is controlled by the right surgeon's hand, and a monopolar fenestrated forceps is controlled by the left surgeon's hand. As usual, the assistant surgeon stands anterior to the patient handling the suction catheter in the space between the three trocars. A suction catheter is not used only to suck fluids but mainly for retraction and exposure of structures. Vascular structures and pulmonary parenchyma are sutured with Sureform 45 Robotic Staplers or with Hem-o-lok robotic clips. Lobectomy or segmentectomy is performed respecting the standard anterior approach (13) to the hilar structures and the fissureless technique (14), whenever possible. At the end of the surgery, a single chest drain toward the apex is placed by the assistant surgeon.

## Results

No intraoperative or perioperative mortality was observed. All procedures were completed with the uniportal approach. We performed 22 lobectomies and 2 segmentectomies; systematic hilar and mediastinal lymph node dissection was accomplished in all patients but 3—the patients with secondary lesions (laryngeal and cervical cancer metastasis). Mean operative time at console including docking was 210 ± 63 min (range 120–350) (Table 2); in the last 10 cases, the operative time was significantly reduced (180 ± 30 min) compared to the first 14 cases (232 ± 72 min) (*p* = 0.02). No patient required blood transfusion, and the mean blood loss was 110 ± 35 ml. No patient required adjunctive administration of drugs to control postoperative pain and no opioid drugs were administered. Furthermore, the mean score of NRS measured on the first postoperative day was 2.6 (±0.6), on the third day was 1.6 (±0.7), and at discharge was 1.3 (±0.4), showing a constant decrease. In four patients (16.7%) minor complications occurred: one prolonged fluid leak (>350 cc/day) was solved spontaneously on day 6, one prolonged air leak was solved spontaneously on day 8, and two atrial fibrillation was treated with pharmacological cardioversion. The mean length of hospital stay was 5.2 ± 1 days (range 3–9). All patients performed an outpatient visit after 30 days from discharge, and in all cases, the functional recovery ranged from satisfactory to good; only two patients were referred to mild local paresthesia.

**Table 2:** Surgical procedures and postoperative results.

Pts	Procedure	Histology	Complications	Hospital stay (days)	Operative time (min)
1	Lower lobectomy	Adenosquamous	0	6	245
2	S6 segmentectomy	Cervix Mtx	Fluid leak	7	240
3	Lower lobectomy	ADC	0	6	315
4	Upper lobectomy	ADC	0	5	290
5	Upper lobectomy	ADC	0	4	350
6	Upper lobectomy	ADC	0	5	330
7	Upper lobectomy	ADC	AF	6	240
8	Upper lobectomy	Squamous cell	AF	6	270
9	Upper lobectomy	ADC	0	4	200
10	Middle lobectomy	Squamous cell	0	4	165
11	S6 segmentectomy	Laryngeal Mtx	0	5	120
12	Lower lobectomy	ADC	0	5	170
13	Lower lobectomy	ADC	0	3	135
14	Upper lobectomy	Squamous cell	0	4	185
15	Lower lobectomy	ADC	0	4	165
16	Upper lobectomy	ADC	Air leak	9	190
17	Upper lobectomy	Laryngeal Mtx	0	6	210
18	Lower lobectomy	Adenosquamous	0	5	180
19	Middle lobectomy	ADC	0	5	190
20	Upper lobectomy	ADC	0	6	185
21	Upper lobectomy	ADC	0	5	210
22	Lower lobectomy	ADC	0	6	135
23	Lower lobectomy	Carcinoid	0	5	215
24	Lower lobectomy	ADC	0	4	125

## Discussion

Typical weaknesses of lung cancer patients have led the surgical community to look for less invasive techniques. Nowadays, U-VATS is the less invasive approach available in thoracic surgery and can be applied to the majority of thoracic surgery procedures, including bronchovascular resection and reconstruction (3, 4). Nevertheless, RATS experience is increasing in many Thoracic Surgery Centers due to well-known advantages such as the 3D vision, lack of physiological tremors, stability of the camera, and a shorter learning curve compared to VATS. However, the RATS technique is always described with three or four incisions plus a utility incision of 4 cm. This is certainly more invasive than the uniportal incision used in U-VATS (15), and uniportal RATS is exclusively a newborn technique that is growing nowadays (10–12).

According to our experience with U-VATS and borrowing from the experience described in the literature (10–12), we started a Uniportal RATS program at the IRCCS “Pascale Foundation” National Cancer Institute of Naples.

The great maneuverability and adaptability of the *da Vinci Xi* robotic system allow many tailored configurations that are of paramount importance using the system through uniportal access. Docking the system in U-RATS is certainly faster than in standard RATS because of the single incision, but it should be performed very carefully to avoid potential fighting between the robotic arms. This can be obtained by keeping a distance of 10 cm between the robotic elbows and a working angle with the chest wall greater than the ones in U-VATS. As the operative arms must cross each other inside the chest (Figure 2), to avoid damage to the ribs, it is mandatory to work as perpendicular to the target as possible. For this reason, differently from U-VATS, in which the instruments enter the chest wall anteriorly with a 45° angle, the surgical incision of U-RATS should be more posterior to allow the arms to work with a mean 70° angle with the chest wall (Figures 1A,B). Due to the intracavity crossing of the instruments, at the touchpad console, the control setting should be modified, changing the arm control, allowing the right master to control the left robotic arm and vice versa. Large movements of masters during surgery should be limited to avoid arms conflict.

Respecting these rules, vessel isolation is easy and always possible without any vessel tension or damage. However, the most time-consuming step of the procedure, in our experience, is represented by vascular stapling due to the dimensional mismatch between robotic staplers and thoracic anatomy. Most of all left upper lobe artery branches or minor right upper lobe branches can be safely managed with the robotic Hem-o-lock clips applier, being smaller and easier to be introduced in the chest. Although using the 45 Sureform Robotic stapler is feasible, it is not easy to approach the vessels and avoid external conflicts between arms and, of course, avoid tension to the vessels. In this scenario, the best equilibrium can be found by balancing the correct stapler angle with a countertraction of the underlining lung parenchyma. The use of the 30 Endowrist curved tip stapler could certainly be helpful but unfortunately it was unavailable in our institute during the study period.

In our opinion, the learning curve of this technique in U-VATS experienced surgeons is quite fast, and we found a significant shortening of the surgical time in the last 10 cases (*p *= 0.02), thus confirming the well-known rapid learning curve of robotic surgery. We did not record any intraoperative complication that needed conversion, but in this case, the switch from U-RATS to U-VATS or thoracotomy is certainly quicker than in standard RATS because removing three arms from a single incision is very fast, without jeopardizing the safety of the patient.

The advantages of RATS have been extensively described in the literature (16–18) and were not the focus of our paper. Still, our experience with both techniques, U-VATS and RATS, showed better postoperative pain control in U-VATS than in RATS patients. Starting from this statement and according to the frailty of our patient population, we decided to evaluate the feasibility and the efficacy of the U-RATS technique, combining the advantages of U-VATS with the well-known advantages of RATS.

The evaluation of the NRS scale was satisfactory in our series and comparable to U-VATS patients in the early postoperative time and 1 month later, confirming that the number of chest incisions is directly related to the postoperative pain, supporting the early recovery.

This technique needs to be tested on a bigger patient population, but in our early experience, we can conclude that U-RATS is certainly safe, feasible, and comparable to U-VATS in terms of postoperative pain results. It remains a time-consuming technique, but the learning curve for skilled U-VATS surgeons is quite fast; furthermore, new suturing devices could simplify the surgical steps through standardization and worldwide spreading of U-RATS.

## Data Availability

The original contributions presented in the study are included in the article/supplementary material; further inquiries can be directed to the corresponding author/s.

## References

[1] Gonzalez-Rivas D Soultanis KM Garcia A Yang K Qing Y Yie L Uniportal video-assisted thoracoscopic lung sparing tracheo-bronchial and carinal sleeve resections. J Thorac Dis. (2020) 12(10):6198–209. 10.21037/jtd.2020.04.05 33209458 PMC7656374

[2] Paradela de la Morena M De La Torre Bravos M Fernandez Prado R Minasyan A Garcia-Perez A Fernandez-Vago L Standardized surgical technique for uniportal video-assisted thoracoscopic lobectomy. Eur J Cardiothorac Surg. (2020) 58(Suppl. 1):i23–33. 10.1093/ejcts/ezaa110 32449910

[3] Mercadante E Alessandrini G Forcella D Melis E Gallina F Facciolo F. Uniportal thoracoscopic left main bronchus resection with new lobar carina reconstruction. Multimed Man Cardiothorac Surg. (2020) 2020. 10.1510/mmcts.2020.039 32633904

[4] Gonzalez-Rivas D Garcia A Chen C Yang Y Jiang L Sekhniaidze D Technical aspects of uniportal video-assisted thoracoscopic double sleeve bronchovascular resections. Eur J Cardiothorac Surg. (2020) 58(Suppl_1):i14–22. 10.1093/ejcts/ezaa037 32083654

[5] Gao Y Abulimiti A He D Ran A Luo D. Comparison of single- and triple-port VATS for lung cancer: a meta-analysis. Open Med (Wars). (2021) 16(1):1228–39. 10.1515/med-2021-0333 34514169 PMC8389499

[6] Li T Xia L Wang J Xu S Sun X Xu M Uniportal versus three-port video-assisted thoracoscopic surgery for non-small cell lung cancer: a retrospective study. Thorac Cancer. (2021) 12(8):1147–53. 10.1111/1759-7714.13882 33586338 PMC8046032

[7] Demos DS Tisol WB. Robotic thoracic lymph node dissection for lung cancer. Video-Assist Thoracic Surg. (2020) 5:17. 10.21037/vats.2020.01.03

[8] Ricciardi S Davini F Zirafa CC Melfi F. From “open” to robotic assisted thoracic surgery: why RATS and not VATS? J Vis Surg. (2018) 4:107. 10.21037/jovs.2018.05.07 29963396 PMC5994439

[9] Oh DS Tisol WB Cesnik L Crosby A Cerfolio RJ. Port strategies for robot-assisted lobectomy by high-volume thoracic surgeons: a nationwide survey. Innovations (Phila). (2019) 14(6):545–52. 10.1177/1556984519883643 31739719

[10] Gonzalez-Rivas D Bosinceanu M Motas N Manolache V. Uniportal robotic-assisted thoracic surgery for lung resections. Eur J Cardiothorac Surg. (2022):ezac410. 10.1093/ejcts/ezac410. [Epub ahead of print]. 35951763

[11] Gonzalez-Rivas D Manolache V Bosinceanu ML Gallego-Poveda J Garcia-Perez A de la Torre M Uniportal pure robotic-assisted thoracic surgery—technical aspects, tips and tricks. Ann Transl Med. (2022). 10.21037/atm-22-1866. [Epub ahead of print]. PMC10477623 37675313

[12] Yang Y Song L Huang J Cheng X Luo Q. A uniportal right upper lobectomy by three-arm robotic-assisted thoracoscopic surgery using the *da Vinci* (Xi) Surgical System in the treatment of early-stage lung cancer. Transl Lung Cancer Res. (2021) 10(3):1571–5. 10.21037/tlcr-21-207 33889530 PMC8044472

[13] Hansen HJ Petersen RH Christensen M. Video-assisted thoracoscopic surgery (VATS) lobectomy using a standardized anterior approach. Surg Endosc. (2011) 25(4):1263–9. 10.1007/s00464-010-1355-9 20927543

[14] Igai H Kamiyoshihara M Yoshikawa R Osawa F Kawatani N Ibe T The efficacy of thoracoscopic fissureless lobectomy in patients with dense fissures. J Thorac Dis. (2016) 8(12):3691–6. 10.21037/jtd.2016.12.58 28149565 PMC5227190

[15] Park BJ Flores RM Rusch VW. Robotic assistance for video-assisted thoracic surgical lobectomy: technique and initial results. J Thorac Cardiovasc Surg. (2006) 131(1):54–9. 10.1016/j.jtcvs.2005.07.031 16399294

[16] Kent MS Hartwig MG Vallières E Abbas AE Cerfolio RJ Dylewski MR Pulmonary open, robotic and thoracoscopic lobectomy (PORTaL) study: an analysis of 5,721 cases. Ann Surg. (2021). 10.1097/SLA.0000000000005115. [Epub ahead of print]. PMC9891268 34534988

[17] Veronesi G Novellis P Voulaz E Alloisio M. Robot-assisted surgery for lung cancer: state of the art and perspectives. Lung Cancer. (2016) 101:28–34. 10.1016/j.lungcan.2016.09.004 27794405

[18] Yang HX Woo KM Sima CS Bains MS Adusumilli PS Huang J Long-term survival based on the surgical approach to lobectomy for clinical stage I nonsmall cell lung cancer: comparison of robotic, video-assisted thoracic surgery, and thoracotomy lobectomy. Ann Surg. (2017) 265(2):431–7. 10.1097/SLA.0000000000001708 28059973 PMC5033685

